# Relative Level of Bacteriophage Multiplication *in vitro* or in Phyllosphere May Not Predict *in planta* Efficacy for Controlling Bacterial Leaf Spot on Tomato Caused by *Xanthomonas perforans*

**DOI:** 10.3389/fmicb.2018.02176

**Published:** 2018-09-18

**Authors:** Botond Balogh, Nguyen Thi Thu Nga, Jeffrey B. Jones

**Affiliations:** ^1^Plant Pathology Department, University of Florida, Gainesville, FL, United States; ^2^Department of Plant Protection, Can Tho University, Can Tho, Vietnam

**Keywords:** bacterial spot of tomato, *Xanthomonas perforans*, *Xanthomonas citri*, citrus canker, biological control

## Abstract

Following analysis of eight phages under *in vitro*, growth chamber and greenhouse conditions with the bacterial spot of tomato pathogen *Xanthomonas perforans*, there was no correlation between disease control efficacy and *in vitro* phage multiplication, *in vitro* bacterial suppression, or *in vivo* phage multiplication in the presence of the host, but there was a low correlation between phage persistence on the leaf surface and disease control. Two of the 8 virulent phages (ΦXv3-21 and ΦXp06-02) were selected for in depth analysis with two *X. perforans* (Xp06-2-1 and Xp17-12) strains. In *in vitro* experiments, phage ΦXv3-21 was equally effective in infecting the two bacterial strains based on efficiency of plating (EOP). Phage ΦXp06-02, on the other hand, had a high EOP on strain Xp06-2-1 but a lower EOP on strain Xp17-12. In several growth chamber experiments, ΦXv3-21 was less effective than phage ΦXp06-02 in reducing disease caused by strain Xp06-2-1, but provided little or no disease control against strain Xp17-12. Interestingly, ΦXp06-02 could multiply to significantly higher levels on the tomato leaf surface than phage ΦXv3-21. The leaf surface appears to be important in terms of the ability of certain bacteriophages to multiply in the presence of the bacterial host. ΦXv3-21, when applied to grapefruit leaves in combination with a bacterial host, was unable to multiply to high levels, whereas on tomato leaflets the phage multiplied exponentially. One plausible explanation is that the leaf surface may be an important factor for attachment of certain phages to their bacterial host.

## Introduction

Bacterial diseases are a major problem on crops in temperate, sub-tropical and tropical environments. Although disease control strategies are available for many bacterial-incited diseases, challenges exist that minimize the ability to control many bacterial pathogens. For most bacterial incited diseases, an integrated management strategy is used and includes plant resistance, biological control, chemical control and various cultural practices to minimize inoculum (Obradovic et al., 2004, 2005).

In Florida, copper based compounds and antibiotics have been used to control bacterial spot of tomato and pepper. Streptomycin, an aminoglycoside antibiotic, was used extensively in the 1950s (Thayer and Stall, 1961). Following its initial use, in a matter of years, strains quickly developed resistance (Thayer and Stall, 1961). As a result, streptomycin no longer was effective in controlling bacterial spot of tomato and pepper and growers returned to using copper based bactericides. Management strategies for fire blight of apple and pear relied on streptomycin for many years (Cooksey, 1990; Manulis et al., 1998). Resistance to this antibiotic was detected in *Erwinia amylovora* strains and was determined to be associated with a plasmid, unlike in the bacterial spot pathogen *Xanthomonas euvesicatoria* in which resistance was associated with spontaneous mutation.

Although copper bactericides have been used extensively, copper resistance was not observed in *X. euvesicatoria* strains until 1983 (Marco and Stall, 1983) and the copper resistance was determined to be associated with a plasmid (Stall et al., 1986). Copper resistance has been found in diverse plant pathogens (Bender and Cooksey, 1986, 1987; Stall et al., 1986; Bender et al., 1990; Lee et al., 1994; Manulis et al., 1998; Basim et al., 1999; Canteros, 1999; Behlau et al., 2011). In most cases, copper resistance has been associated with plasmids (Bender and Cooksey, 1986, 1987; Stall et al., 1986; Bender et al., 1990; Behlau et al., 2011) and to a lesser extent copper resistance genes are associated with the chromosome (Lee et al., 1994; Basim et al., 1999; Behlau et al., 2017). Copper-tolerant strains of *X. euvesicatoria* were shown to be sensitive to copper bactericides when mixed with ethylene-bis-dithiocarbamates (Marco and Stall, 1983); unfortunately, during optimal disease conditions, copper-mancozeb has not been consistently effective in controlling bacterial spot nor in increasing yield (Jones and Jones, 1985; Obradovic et al., 2004).

Biological control as an alternative disease control strategy for bacterial diseases on tomato has focused extensively on using non-pathogenic microorganisms including Hrp-strains (pathogenic strains mutated in the Hrp-region and rendered non-pathogenic) of pathogens to suppress foliar or root pathogens in order to reduce disease (Frey et al., 1994; Liu, 1998; Wilson et al., 2002; Byrne et al., 2005; Ji et al., 2006; Hert, 2007; Jones et al., 2007; Obradovic et al., 2008; Iriarte et al., 2012). Plant growth-promoting rhizobacteria (PGPR) have been extensively tested for suppressing disease as a result of induction of plant defense responses in the plant (Obradovic et al., 2005; Ji et al., 2006). These approaches have achieved varying levels of success. Another approach consisted of using bacteriocin-producing strains that are inhibitory to pathogenic strains of a closely related organism (Chen and Echandi, 1984; Hert, 2007; Hert et al., 2009).

Bacteriophages have also been used as biological control agents (Jones et al., 2007). Mallmann and Hemstreet (1924) determined that liquid filtrate from black rot infected cabbage tissue inhibited growth of *X. campestris in vitro*. As research on bacteriophages progressed, their presence in other plant tissue was determined. Coons and Kotila (1925) isolated phages from various sources, such as rotting carrots, soil, and river water, that were inhibitory to *Erwinia carotovora* subsp. *carotovora* and *Agrobacterium tumefaciens*. Furthermore, they isolated phages from soil samples associated with “black leg” symptoms on potato (Kotila and Coons, 1925). The isolated phages inhibited the disease causal agent *E. carotovora* subsp. *atroseptica* and when co-inoculated with the pathogen prevented rotting of potato tubers. The first field trials were conducted by Thomas (1935) against Stewart’s wilt of corn. In that study corn seeds infested with the pathogen *Pantoea stewartii* were treated with phages isolated from diseased plant material. This seed treatment was quite effective and resulted in a reduction in disease incidence. In that study treatment of *P. stewartii*-infected corn seed resulted in a reduction in disease incidence from 18 to 1.4%. Moore (1926) proposed using bacteriophages for disease control of bacterial plant pathogens. Over the years they have been used for several plant–bacterium pathosystems to demonstrate the efficacy of phages for disease control (Civerolo and Keil, 1969; Tanaka et al., 1990; Zaccardelli et al., 1992; Saccardi et al., 1993; Flaherty et al., 2000).

Bacteriophages offer an alternative to conventional management strategies for controlling plant diseases caused by bacterial pathogens (Civerolo, 1973; Tanaka et al., 1990; Gill et al., 2003; Jackson et al., 2004; Obradovic et al., 2005; Jones et al., 2006; Jones et al., 2007; Balogh et al., 2008; Obradovic et al., 2008; Fujiwara et al., 2011; Gašic et al., 2011; Murugaiyan et al., 2011). Although various studies showed that phage-therapy was plausible for controlling plant pathogenic bacteria associated with several systems, Okabe and Goto (1963) concluded that phages were not an effective control strategy. Various concerns as to their limitations have been discussed. Their narrow spectrum of activity against specific bacterial species is a concern in comparison to antibacterial materials, such as antibiotics, which have broad spectrum activity (Summers, 2005). Along with the narrow spectrum of activity, the probability that bacteria become resistant to individual phages via mutation is a real concern. Katznelson (1937) observed this, as well as Okabe and Goto (1963), and Vidaver (1976). The latter two viewed the possibility of mutation as a major impediment for use as a control strategy.

The environment in the phyllosphere is deleterious to phage resulting in precipitous declines in bacteriophage population (Civerolo and Keil, 1969; McNeil et al., 2001; Balogh, 2002; Balogh et al., 2003). This short-lived persistence on plant leaf surfaces is the major limiting factor for phage therapy in the phyllosphere. In the phyllosphere phages are exposed to deleterious factors, with their viability plummeting in a very short period of time. These factors include sunlight irradiation, especially in the UV-A and -B regions, ambient temperature, desiccation and exposure to certain chemical pesticides, such as copper-based bactericides that are commonly used for bacterial disease management (Iriarte et al., 2007). Of these factors, UV was found to be the most deleterious factor, especially in the early afternoon hours.

In biological control, maintaining high populations of biocontrol agents in relatively close proximity to the target bacterium is critical to their success (Johnson, 1994). Persistence of phage in the phyllosphere and rhizosphere has been a major concern, given that phage therapy necessitates high densities of phage exist in close proximity to the target pathogen (Gill and Abedon, 2003). Phages must be present at a certain titer (i.e., threshold titer) relative to the target bacterium; at concentrations below this, phages will have a minimal impact on disease control. Balogh (2002) demonstrated that a threshold of 10^6^ or 10^8^ PFU/ml of *X. perforans* specific phage provided similar control of bacterial spot on tomatoes inoculated with 10^8^ cfu/ml of *Xanthomonas perforans*, but phage applied at 10^4^ PFU/ml was ineffective. Given the inability to persist for long periods on leaf surfaces and the requirement for high phage populations, long-term survival of phage on leaf surfaces requires different strategies for maintaining high phage concentrations.

Various approaches have been used to increase persistence in the phyllosphere. One approach, which involved applying phage to plants in the evening, was effective for maintaining high phage concentrations on the leaf surface for an extended period of time and was associated with improved disease control (Balogh et al., 2003). A second approach used was to mix phage suspensions with various compounds (formulations) to extend bacteriophage persistence on leaf surfaces (Balogh et al., 2003; Iriarte et al., 2007). Although formulations were identified and shown to be effective, phage levels still plummeted below detectable levels on leaves that were free of the target bacterium (Balogh, 2002; Balogh et al., 2003).

A third approach tested for enhancing persistence was to increase phage populations in the phyllosphere by multiplying on bacterial hosts that are not pathogens or pathogens that are impaired in virulence. This ability could potentially be used if phages are applied into an environment where a phage-sensitive bacterium is present, or where the phages and bacterial host are delivered together. On leaf surfaces, phages persist at high populations on infected leaves where there are high concentrations of the bacterial pathogen (phage host) than on leaf surfaces without the host (Iriarte et al., 2012). Boulè et al. (2011) and Svircev et al. (2005) utilized a different strategy in which a non-pathogen (*Pantoea agglomerans*) host to the phage was applied to apple flower blossoms to enhance phage populations that in turn would be present to infect and reduce population of *Erwinia amylovora*.

We have observed that bacteriophages behave differently on leaf surfaces than *in vitro*. Therefore, we present information that predicting the ability to control the bacterial pathogen on leaf surfaces based solely on *in vitro* results is not sufficient to predict efficacy *in planta*. In this study our goal was to monitor the population dynamics of bacteriophages and their interaction with their host bacterium in the phyllosphere. More specifically, we wanted to answer the following questions: Is there a difference between the ability of different phages to multiply in the phyllosphere? If so, is there a connection between success in *in planta* multiplication and disease control ability? Does the nature of the phyllosphere itself influence phage-bacterium interaction (i.e., do some plants support phage multiplication better than others)? And lastly, can we learn something from these studies that could be used for improving phage therapy for plant protection?

## Materials and Methods

### Bacterial Strains and Bacteriophages

Bacterial strains and bacteriophages used in this study are listed in **Supplementary Table 2**.

### Interaction of *Xanthomonas citri* subsp. *citri* and Its Bacteriophages on Grapefruit Foliage in the Greenhouse

#### Disease Control Studies

Two experiments were carried out at the greenhouse of the Department of Agriculture & Consumer Services, Division of Plant Industry, citrus canker 84 quarantine facility. Duncan grapefruit plants were heavily pruned and fertilized to induce the simultaneous production of a new flush that is susceptible to citrus canker infection. Three weeks later, uniform, new foliage emerged and was treated with one of three different single-phage suspensions (ΦXV3-21, ΦXaacF1, or ccΦ19-1, 5 × 10^9^ PFU/mL, at 50 mL/plant) or with sterilized tap water. The treatments were applied in the evening using a hand-held sprayer, and then the plants were placed in white plastic bags. The following morning the bags were removed from the plants, and the plants were spray-inoculated with a bacterial suspension of *X. citri* strain Xac65 adjusted to 1 × 10^6^ CFU/mL at the rate of 50 mL/plant. After inoculation the plants were placed inside the bags for an additional 24 h of high moisture. After removal from the bags and after the foliage was allowed to dry the plants were arranged in a completely randomized pattern on a greenhouse bench. The disease was assessed 3–4 weeks after inoculation. In the first experiment the phage suspensions were prepared by diluting high titer lysates, but because of concerns that the presence of nutrient broth in the phage lysate may contribute to increased disease severity, in the second experiment the phage lysates were concentrated in order to remove nutrient broth. The ratio of diseased leaf surface area was estimated using the Horsfall-Barratt (HB) scale (12). The HB values were converted to estimated mean percentages by using the Elanco Conversion Tables for Horsfall-Barratt Rating Numbers (Elanco Products Co., Indianapolis, IN, United States). Analysis of variance (ANOVA) and subsequent separation of sample means by Student-Newman-Keuls means comparison test (*P* = 0.05) was carried out using the software package ARM Revision 2018.3.

#### Population Dynamics Studies on Grapefruit and Tomato

In order to determine if bacteriophages ΦXV3-21, ΦXaacF1, and ccΦ19-1 are able to multiply on the grapefruit foliage in the presence of their host, Xac65, “Duncan” grapefruit plants were sprayed with a mixture of the three phages at low concentration (5 × 10^6^ PFU/mL; 50 mL/plant), immediately followed by application of the bacterial suspension at a much higher concentration (1 × 10^8^ cfu/mL) or with sterilized tap water. Phage populations were monitored by removing three leaves 9 h after application, recovering the phages from the leaves and determining concentrations. In order to determine populations of the individual phages, the leaf washes were plated on three Xac strains that specifically detected each of the three phages. Each strain was only sensitive to one of three phages. Strain Xac41 was used for specific detection of ΦXaacF1, Xac15 for ccΦ19-1 and Xac30 for ΦXv3-21. Tomato trials were conducted using the same methodology, using “Bonny Best” tomato cultivar.

### Interaction of *Xanthomonas perforans* and Its Phages *in vitro* and *in planta*

#### Greenhouse Disease Control Trials

The objective of these trials was to evaluate the efficacy of 8 individual phages to reduce tomato bacterial spot disease caused by *X. perforans* strain Xp06-2-1. Young “Bonny Best” tomato plants were dipped in phage suspension (5 × 10^7^ PFU/mL), and 2 h later spray-inoculated with Xp06-2-1 (5 × 10^6^ CFU/mL). After inoculation the plants were placed in plastic bags, and kept in a growth chamber for 36 h. Afterward, they were transported into a greenhouse and taken out of the bags. Disease severity was assessed 10–14 days after inoculation.

#### *In planta* Phage Persistence and Multiplication Assay

Eight *Xanthomonas perforans* phages were evaluated for their ability to persist and to multiply on the tomato phyllosphere. Plants were dipped in phage suspensions (5 × 10^6^ PFU/ml) and then spayed with water or a bacterial suspension of *X. perforans* strain Xp06-2-1 adjusted to 10^8^ CFU/mL. Phage titer was determined at the beginning and after overnight incubation (16 h). Plants were bagged throughout this experiment.

#### *In vitro* Phage Multiplication and Bacterial-Growth-Suppression Assays

Individual phages, at 10^7^ PFU/mL were incubated for 16 h with a bacterial suspension of *X. perforans* strain Xp06-2-1 adjusted to 10^8^ CFU/mL. The final and original phage titers were compared to determine the amount of phage multiplication. The final bacterial concentrations were compared between phage-infected and non-infected bacterial cultures to determine the effect of individual phages in reducing bacterial populations.

## Results

### Disease Control Trials on Grapefruit

Three bacteriophages, ΦXv3-21, ccΦ19-1, and ΦXaacF1, all able to lyse *X. citri* subsp. *citri* strain Xac65, were evaluated for their ability to reduce citrus canker incited by Xac65 in grapefruit in two greenhouse trials. Of the three phages, only ΦXaacF1 resulted in significant disease reduction: 58 and 69% reduction in the two experiments (**Table 1**). Plants treated with ccΦ19-1 had a slight but non-significant disease reduction in both trials (10 and 31%), whereas ΦXv3-21 appeared to increase the disease severity slightly (-31 and -21%).

**Table 1:** Comparative efficacy of three bacteriophages, applied as foliar preventative sprays, on citrus canker disease development, incited by phage-sensitive *Xanthomonas citri* subsp. *citri* strain Xac65, as measured by disease severity.

	Mean citrus canker severity [%]				Reduction in severity - Abbott [%]	
	Trial 1 – 25 DAT^x,y^		Trial 2 – 16 DAT		Trial 1 – 25 DAT	Trial 2 – 16 DAT
Untreated	44.5	a	21.7	ab	0.0	0.0
ΦXv3-21	58.4	a	26.2	a	-31.2	-20.6
ccΦ19-1	40.0	a	14.9	ab	10.1	31.4
ΦXaacF1	18.8	b	6.8	b	57.9	68.8
p(F)^z^	0.0200		0.0395			

*^z^p(F) = Probability that there are no differences in treatment means according to analysis of variance.*

*^y^Means within the same column followed by the same letter are not significantly different (P = 0.05, Student-Newman-Keuls).*

*^x^DAT = days after treatment.*

### Interaction of Three Bacteriophages and Their Host, *X. citri* subsp. *citri* Strain Xac65 in the Grapefruit Phyllosphere

A mixture of the above mentioned three bacteriophages was sprayed on grapefruit plants, which were immediately sprayed with a suspension of Xac65 or tap water, and the phage populations were monitored over the next 9 h. All three bacteriophages declined rapidly after application to the grapefruit leaves without host bacterium being present with more than a 95% reduction in their populations over the 9 h period (**Table 2**). However, in the presence of the host, Xac65, all three phages showed some signs of multiplication in at least one of the two experiments, based on their ability to maintain higher populations after a 9 h period compared to phages without a bacterial host. However, their responses varied greatly. ΦXv3-21 and ccΦ19-1 populations dropped even in the presence of Xac65, although not as quickly as without their host (80% reduction with host vs. 99% without it for ΦXv3-21 in experiment 2, and 89 vs. 97% for ccΦ19-1). ΦXaacF1, on the other hand, actually increased in numbers in the presence of its host: 95% reduction vs. 30% increase in exp. 1, and 94% reduction vs. 239% increase in exp. 2. Altogether, ΦXaacF1 persisted better than the other two phages without a host and multiplied to a limited extent in the presence of the host.

**Table 2:** Population dynamics of three citriphages in the grapefruit phyllosphere in the presence or absence of their host bacterium, *Xanthomonas citri* subsp. *citri* strain Xac65.

*Grapefruit*												
	**Experiment 1**						**Experiment 2**					
Bacteriophage populations on grapefruit	ccΦ19-1^z^ [Xac15]		ΦXv3-21 [Xac30]		ΦXaacF1 [Xac41]		ccΦ19-1 [Xac15]		ΦXv3-21 [Xac30]		ΦXaacF1 [Xac41]	

**Phage populations recovered from the leaves [log10 pfu/leaf]^y^**												

0 h	3.23	a	3.91	a	4.29	a	3.90	a	4.83	a	4.21	b
9 h| without host	0.38	b	2.14	b	2.91	b	1.51	b	2.66	c	2.93	c
9 h| with host	0.00	b	1.4	b	4.19	a	3.09	a	3.78	b	4.72	a

**Relative changes in phage populations over time [% increase vs. 0 h]**												

9 h| without host	-100		-98		-95		-99		-97		-94	
9 h| with host	-100		-99		30		-80		-89		239	

**Relative frequency in residual phage population mixture [%]**												

9 h| without host	4		29		67		12		13		75	
9 h| with host	0		1		99		7		4		89	

*^z^Bacteriophages were exclusively detected on the bacterial stains shown in parentheses.*

*^y^Means within the same column followed by the same letter are not significantly different (P = 0.05, Student-Newman-Keuls).*

Phages were applied in equal concentration, so the original ratio of the three phages was 33/33/33. In the absence of the host, ΦXaacF1 came to dominate the declining phage population and made up 67–75% of the recoverable phage population after 9 h of incubation (**Table 2**). In the presence of the host, ΦXaacF1 dominated even more constituting 89–99% of the total phage population. Of these three phages, only ΦXaacF1, which was most successful in multiplying on its host bacterium on the plant surface, was able to control citrus canker disease progress (**Table 1**).

### Interaction of Three Bacteriophages With *Xanthomonas citri* subsp. *citri* Strain Xac65 in the Tomato Phyllosphere

We speculated that the waxy grapefruit leaf provides a less than ideal environment for the bacteriophages. Thus we decided to look at the interaction of the same three phages and their bacterial host in a different environment, the phyllosphere of the incompatible host, tomato.

Without the host all three phages deteriorated in a 9-h period, but to a much lesser degree than in the grapefruit leaves, 24–66% population reduction (**Table 3**). ΦXaacF1 suffered the worst decline on tomato in both experiments, but still 34 and 43% of its populations were recovered after 9 h (**Table 3**). Additionally, in tomato all three phages actually increased in numbers in the presence Xac65. ΦXv3-21 produced the slightest (and non-significant) increase: 23 and 78% increase in two experiments. ccΦ19-1 populations increased by 82 and 327%. ΦXaacF1 had a spectacular 169 and 301-fold population increase in experiments 1 and 2, respectively. These results suggest that tomato provides a much better environment for the phages. As for the composition of the phage mixture: in the absence of the hosts, the ratio stayed close to the originally applied 33:33:33 ratio. But due to the unequal capacities for multiplication in the presence of the host, ΦXaacF1 came to dominate the population providing 97 and 99% of the total in the two experiments.

**Table 3:** Population dynamics of three citriphages in the tomato phyllosphere in the presence or absence of their host bacterium *Xanthomonas citri* subsp. *citri* strain Xac65.

*Tomato*												
	**Experiment 1**						**Experiment 2**					
Bacteriophage populations on tomato	ccΦ19-1^z^ [Xac15]		ΦXv3-21 [Xac30]		ΦXaacF1 [Xac41]		ccΦ19-1 [Xac15]		ΦXv3-21 [Xac30]		ΦXaacF1 [Xac41]	

**Phage populations recovered from the leaves [log10 pfu/leaflet]^y^**												

0 h	6.10	b	5.69	ab	5.29	b	6.28	ab	5.73	-	5.35	b
9 h| without host	5.98	b	5.29	b	4.82	b	5.99	b	5.50	-	4.98	c
9 h| with host	6.73	a	5.94	a	7.52	a	6.54	a	5.82	-	7.83	a

**Relative changes in phage populations over time [% increase vs. 0 h]**												

9 h| without host	-24		-60		-66		-49		-41		-57	
9 h| with host	327		78		16,882		82		23		30,100	

**Relative frequency in residual phage population mixture [%]**												

9 h| without host	49		27		24		33		38		28	
9 h| with host	2		1		97		1		0		99	

*^z^Bacteriophages were exclusively detected on the bacterial stains shown in parentheses.*

*^y^Means within the same column followed by the same letter are not significantly different (P = 0.05, Student-Newman-Keuls).*

### Interaction of Bacteriophages and Their Host, *Xanthomonas perforans*, *in vitro* and in the Phyllosphere

Two phages, ΦXp06-02 and ΦXv3-21, were chosen for the following experiments. ΦXp06-02 is a narrow host-range phage isolated from tomato bacterial spot lesions (**Supplementary Table 1**). Phage ΦXv3-21, on the other hand, has broad host range, and is able to lyse a number of *X. perforans* strains (**Supplementary Table 1**) as well as other xanthomonads, such as *X. citri* subsp. *citri* strains (**Table 1**). In greenhouse tests, ΦXp06-02 was more effective in controlling tomato bacterial spot than ΦXv3-21 (**Table 5**). Our aim was to set up a situation in which the two phages are interacting with a strain that both can lyse; and a strain on which ΦXp06-02 is compromised. Xp06-2-1 was chosen as the sensitive strain and Xp17-12 as the strain on which ΦXv3-21 was only effective based on *in vitro* activity. Phage ΦXv3-21 had similar efficiency of plating (EOP) on both bacterial strains (meaning that they were equally successful in infecting both strains). ΦXp06-02 on the other hand, had about a 5% EOP on Xp17-12 compared to on Xp06-2-1 (meaning that it was largely unsuccessful in infecting/lysing this strain). ΦXv3-21 provided slight, but mostly a significant level of control against both strains, Xp06-2-1 and Xp17-12, whereas ΦXp06-02 provided excellent control of Xp06-2-1 and slight-to-no control of Xp17-12 (**Table 4**). The fact that ΦXp06-02 was effective against Xp06-2-1, but not Xp17-12, was similar to the EOP results.

**Table 4:** Effect of phage treatments on tomato bacterial spot disease severity, caused by *Xanthomonas perforans* strains (Xp06-2-1 or Xp17-12), as measured by the Horsfall-Barratt scale.

	**Horsfall-Barratt Ratings [1–12]**					
	**Experiment 1**		**Experiment 2**		**Experiment 3**	
Treatment∖pathogen	Xp06-2-1^z^	Xp17-12	Xp06-2-1	Xp17-12	Xp06-2-1	Xp17-12
Untreated	4.03 a	4.00 a	4.57 a	4.69 a	4.35 a	4.89 a
ΦXv3-21	3.33 ab	3.00 b	3.61 b	4.03 b	3.42 b	3.72 b
ΦXp06-02	3.00 b	3.81 ab	2.42 c	3.95 b	2.36 c	4.22 ab

*^z^Means within the same column followed by the same letter are not significantly different (P = 0.05, Student-Newman-Keuls).*

**FIGURE 1:**
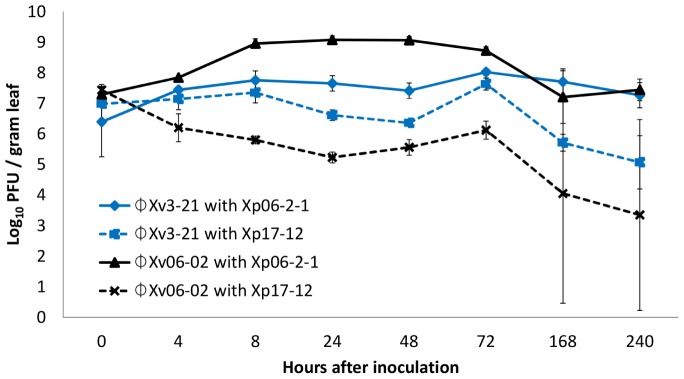
Populations of phages ΦXv3-21 and ΦXp06-02 in the tomato phyllosphere in the presence of *Xanthomonas perforans* strains Xp06-21 or Xp17-12.

The interaction of these two phages with these two *X. perforans* strains in the phyllosphere was also assessed (**Figure 1**). ΦXp06-02 populations increased to significantly higher numbers in the tomato phyllosphere in the presence of Xp06-2-1 cells. However, in the presence of Xp17-12 its populations plummeted, suggesting that it was not able to multiply on this strain. ΦXv3-21 populations were intermediate, but this phage had slightly higher populations on Xp06-2-1 than on Xp17-12. In essence, in this system, the phage population size correlated with disease control efficacy: the ΦXp06-02/Xp06-2-1 interaction resulted in the highest phage population on tomato, and this was also the interaction with the lowest disease severity. On the other hand, the ΦXp06-02/Xp17-12 had the lowest phage populations and the worst disease control.

### *Xanthomonas perforans* Phages – Correlation of Disease Control Efficacy and *in vitro*/*in vivo* Properties

**FIGURE 2:**
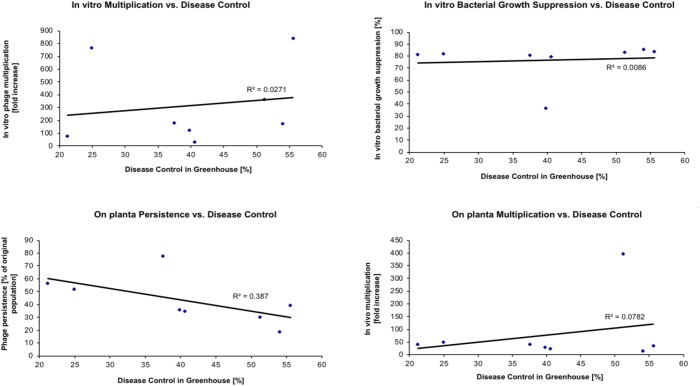
Relationship between phage characteristics and their disease control efficacy in greenhouse. Phages listed in **Table 5** were used in this study with *X. perforans* strain Xp06-2-1. The identity of the specific phage can be determined based on the disease control values in **Table 5**.

The objective of the next set of experiments was to determine if some measurable *in vitro* or *in vivo* phage properties could be used to predict the actual disease control capacity of individual phages. Eight phages were evaluated for their ability to control tomato bacterial spot disease on “Bonny Best” tomato plants caused by *X. perforans* strain Xp06-2-1 under greenhouse conditions (**Table 5**). The following four properties were determined for each phage: (1) the efficacy of multiplying *in vitro* on Xp06-2-1 in liquid culture (i.e., how easy is it to grow the phage on the target bacterium); (2) the efficacy of suppressing Xp06-2-1 growth *in vitro* in liquid culture; (3) the ability to persist *in planta* on “Bonny Best” plants without the presence of a host bacterium; and (4) the ability to multiply *in planta* on “Bonny Best” plants in the presence of Xp06-2-1. These properties were correlated with the measured disease control efficacy (**Figure 2**), and all but ability to persist on the leaf surface showed any apparent correlation with it (i.e., none of these properties had predictive power for the actual disease control activity of the phage). Persistence on the leaf surface had a low correlation with ability to control disease.

**Table 5:** Efficacy of 8 *Xanthomonas perforans*-specific bacteriophages in reducing disease severity of bacterial spot on tomato caused by *X. perforans* strain Xp06-2-1.

	**Disease control [Abbott %]**			
	Trial 1	Trial 2	Trial 3	*Average*
ΦXv3-1	17	48	-2	***21***
ΦXp06-01	5	40	30	***25***
ΦXv3-3	29	48	36	***38***
ΦXv3-21	43	49	27	***40***
ΦXacm2004-11	40	41	na	***41***
ΦXp06-04	58	52	44	***51***
ΦXp06-02	53	62	47	***54***
ΦXv3-16-1h	54	60	53	***56***

## Discussion

In a previous study we demonstrated that disease control efficacy increases if phages persist longer in the target environment, the phyllosphere (Balogh et al., 2003). Several approaches were used to increase longevity including the use of protective formulations, sunlight avoidance or use of propagating hosts (Balogh, 2002; Balogh et al., 2003; Iriarte et al., 2007, 2012). Basically the net change in phage populations is the product of two opposing forces: an increase due to multiplication on host bacterium and a decrease due to many different factors including sunlight irradiation, desiccation and rain-leaching (Balogh et al., 2003; Iriarte et al., 2007). Thus, increasing overall longevity of phages requires protecting the bacteriophage against harmful factors and helping in multiplication.

Our intent was to demonstrate that multiplication on leaf surfaces is important for disease control and that the traditional *in vitro* plaque assays are not sufficient in determining what phages need to be used, because they represent an ideal situation for phage attachment that does not exist in the harsh reality of plant surfaces. We demonstrated that EOP is not the only factor that is required for determining the ability of phage to persist on leaf surfaces when the bacterial host is present. We also showed with two different phages which had similar high EOPs, only one multiplied to high concentrations on tomato leaves while the other dropped to low levels (**Figure 1**). Therefore, predicting the ability to multiply on leaf surfaces cannot be determined strictly based on EOP as it is only a good predictor for those phages-bacterial host combinations where the EOP is low.

We also similarly demonstrated on grapefruit leaves that phage populations increased with certain phage-host combinations and not with others. Interestingly ΦXv-3-21 multiplied on tomato leaflets when the bacterial host was present, but gradually declined on grapefruit leaves with or without a bacterial host. Possibly the leaf surface may be a factor for multiplication of certain phages or perhaps the bacterial host was the contributing factor. Neither one can be ruled out as two different bacterial hosts were used.

We demonstrated that if host bacterium is present on leaves some phages can persist much better. Furthermore, based on these limited tests, it appears that phages that are more successful in multiplying in the phyllosphere environment and are able to keep up higher populations and persist longer as long as their host is present, are able to provide effective disease suppression (**Table 4**). It was also apparent based on these studies that bacterial populations were not affected by the presence of the phage. So using propagating bacteria to maintain phages in the phyllosphere appears to be a promising approach that is worth pursuing further. Actually, it is possible to imagine that future phage therapy products will not only include a mixture of phages, but also one or more propagating bacteria.

And what kind of bacterium would be feasible to maintain phages in the phyllosphere? It should be able to colonize the target plant well enough to build up and maintain sufficient populations, while not causing any adverse effects on plant development. It could be an avirulent strain of the target pathogen (as explored by Tanaka et al. (1990) with *Ralstonia solanacearum*) or a non-pathogenic relative of the target organism (as explored by Svircev et al. with *Erwinia amylovora* and *Pantoea agglomerans*). In case of *Xanthomonas* spp., however, these approaches may not work: although avirulent mutants of *X. perforans* are able to colonize tomato plants successfully (Iriarte et al., 2007) phages are not able to persist at high concentrations. Furthermore, non-pathogenic xanthomonads are not found frequently as epiphytes (personal observations).

It is also interesting to consider if bacterial populations were not affected by the presence of the phage, how does phage therapy reduce disease? In the case of the citrus canker pathosystem, the only one of these three phages that could control citrus canker disease progress, ΦXaacF1, was most successful in multiplying on its host bacterium on the plant surface, and was the only one able to actually increase its populations in the phyllosphere. Interestingly, it appeared during electron microscopic studies that ΦXaacF1 was able to attach to Xac65 in deionized water, while ΦXv3-21 and ccΦ19-1 could only do it in nutrient broth, a complex medium (Balogh, personal observation). Consequently, it seems possible that ΦXaacF1 does not require any metal ion cofactors for attachment as many other phages do (including ΦXv3-21 and ccΦ19-1), and because of this it is readily able to multiply in the low ionic phyllosphere environment. And this would not be a surprise, since ΦXaacF1 was isolated from citrus leaf lesions (Balogh, 2006), and so it probably had an evolutionary pressure to be less needy.

Whatever the reason for a phage’s ability to successfully multiply in the phyllosphere, it is likely that phages originating from the phyllosphere will be in general better adapted to this environment. And if the above connection is true, meaning better phyllosphere multiplication translates to better disease control, then phyllosphere phages are likely to be better biocontrol agents than other phages. Also, if multiplication is important for disease control then the traditional *in vitro* plaque assays are not sufficient in determining what phages need to be used, because they represent an ideal situation for phage attachment that does not exist in the harsh reality of plant surfaces. Our results also showed that the success of phage multiplication depends on the plant, as well. In this case two phages that were unable to multiply on grapefruit sufficiently to at least maintain current population levels were able to do so on tomato. And the third phage, which increased in numbers on grapefruit, produced a much bigger population increase on tomato (single vs. triple digit increase in grapefruit and tomato, respectively). This finding implies that phage therapy will be more successful on some plants than on others.

## Author Contributions

BB and JJ conceived the project. All authors oversaw the experiments, performed data analyses and interpreted them, and wrote and approved the final manuscript.

## Conflict of Interest Statement

The authors declare that the research was conducted in the absence of any commercial or financial relationships that could be construed as a potential conflict of interest. The reviewer GW and handling Editor declared their shared affiliation at the time of review.
