# Corrigendum: Relative Level of Bacteriophage Multiplication *in vitro* or in Phyllosphere May Not Predict *in planta* Efficacy for Controlling Bacterial Leaf Spot on Tomato Caused by *Xanthomonas perforans*

**DOI:** 10.3389/fmicb.2018.02647

**Published:** 2018-10-29

**Authors:** Botond Balogh, Nguyen Thi Thu Nga, Jeffrey B. Jones

**Affiliations:** ^1^Plant Pathology Department, University of Florida, Gainesville, FL, United States; ^2^Department of Plant Protection, Can Tho University, Can Tho, Vietnam

**Keywords:** bacterial spot of tomato, *Xanthomonas perforans*, *Xanthomonas citri*, citrus canker, biological control

In the original article, there was a mistake in Figure 1 as published. The author and the Frontiers Production Office published Figure 2 as Figure 1 in error. The missing Figure 1 appears below.

**Figure 1:**
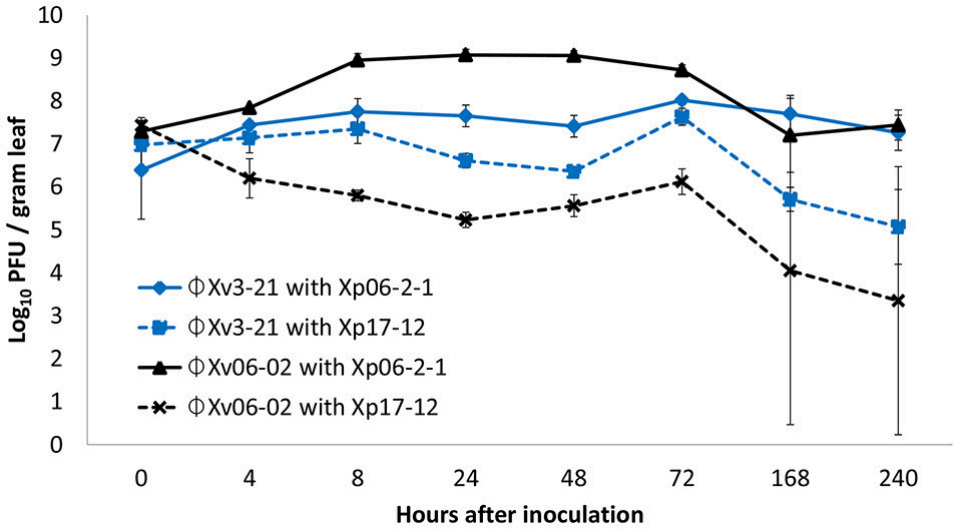
Populations of phages ΦXv3-21 and ΦXp06-02 in the tomato phyllosphere in the presence of *Xanthomonas perforans* strains Xp06-21 or Xp17-12.

In addition, there was an error in the affiliations for author BB. Nichino Europe, Co., Ltd., Cambridge, United Kingdom is the author's current affiliation and not the one held at the time this research was conducted. Therefore, the affiliation list has been updated to reflect this and Nichino Europe added as the present address.

The authors and the Frontiers Production Office apologize for these errors and state that they do not change the scientific conclusions of the article in any way. The original article has been updated.

## Conflict of interest statement

The authors declare that the research was conducted in the absence of any commercial or financial relationships that could be construed as a potential conflict of interest.

