# Supplementary material for: Relative Level of Bacteriophage Multiplication in vitro or in Phyllosphere May Not Predict in planta Efficacy for Controlling Bacterial Leaf Spot on Tomato Caused by Xanthomonas perforans
Source: Front Microbiol. 2018 Sep 18;9:2176. doi: 10.3389/fmicb.2018.02176 (PMC6157332; doi:10.3389/fmicb.2018.02176)
Supplement: Supplementary file 1 [file Table_1.DOCX]

|  |  |
| --- | --- |

Supplementary table 1. Host range profile of bacteriophages used in the tomato bacterial spot experiments.

| Phage \ bacterial strain | Xp62-1 | Xp64-1 | Xp68-1 | Xp6-20-1 | 91-118 | 91-106 | MME |
| --- | --- | --- | --- | --- | --- | --- | --- |
| ΦXacm2004-11 | +^z^ | + | + | + | + | + | + |
| ΦXv3-21 | + | + | + | + | + | + | + |
| ΦXv3-16-1h | + | + | + | + | + | + | + |
| ΦXv3-1 | + | - | - | - | + | + | - |
| ΦXp06-02 | + | - | - | - | + | - | - |
| ΦXv3-3 | + | - | - | - | - | + | - |
| ΦXp06-01 | + | - | - | + | - | - | - |
| ΦXp06-04 | + | - | - | - | - | - | - |
| ΦXv3-18 | + | - | - | - | - | - | - |

^z^ +=lysis; - = no lysis
