# Supplementary material for: Relative Level of Bacteriophage Multiplication in vitro or in Phyllosphere May Not Predict in planta Efficacy for Controlling Bacterial Leaf Spot on Tomato Caused by Xanthomonas perforans
Source: Front Microbiol. 2018 Sep 18;9:2176. doi: 10.3389/fmicb.2018.02176 (PMC6157332; doi:10.3389/fmicb.2018.02176)
Supplement: Supplementary file 2 [file Table_2.docx]

Supplementary table 2. Bacterial strains and bacteriophages used in this study.

| Strain Designation | Species | Other Information |
| --- | --- | --- |
|  | | |
| *Bacterial strains* | | |
| Xp06-2-1 | *Xanthomonas perforans* | isolated from tomato in Florida |
| Xp06-4-1 | *Xanthomonas perforans* | isolated from tomato in Florida |
| Xp06-8-1 | *Xanthomonas perforans* | isolated from tomato in Florida |
| Xp06-20-1 | *Xanthomonas perforans* | isolated from tomato in Florida |
| 91-118 | *Xanthomonas perforans* | isolated from tomato in Florida |
| 91-106 | *Xanthomonas euvesicatoria* | isolated from tomato in Florida |
| MME | *Xanthomonas vesicatoria* |  |
| Xp17-12 | *Xanthomonas perforans* | isolated from tomato in Florida |
| Xac15 | *Xanthomonas citri* subsp. *citri* | isolated from grapefruit in Florida, alt. ID: W4 |
| Xac30 | *Xanthomonas citri* subsp. *citri* | isolated from grapefruit in Florida, alt. ID: XI2001-00098 |
| Xac41 | *Xanthomonas citri* subsp. *citri* | isolated from grapefruit in Florida, alt. ID: XS2000-00060 |
| Xac65 | *Xanthomonas citri* subsp. *citri* | isolated from grapefruit in Florida, alt. ID: XI2000-00120 |
|  |  |  |
| *Bacteriophages* | | |
| ΦXacm2004-11 | | isolated from citrus bacterial spot lesion in Florida |
| ΦXv3-21 |  | received from Dr. L. E. Jackson |
| ΦXv3-16-1h |  | received from Dr. L. E. Jackson |
| ΦXv3-1 |  | received from Dr. L. E. Jackson |
| ΦXp06-02 |  | from tomato bacterial spot lesion in Florida |
| ΦXv3-3 |  | received from Dr. L. E. Jackson |
| ΦXp06-01 |  | from tomato bacterial spot lesion in Florida |
| ΦXp06-04 |  | from tomato bacterial spot lesion in Florida |
| ΦXv3-18 |  | received from Dr. L. E. Jackson |
| ccΦ19-1 |  | received from Dr. L. E. Jackson |
| ΦXaacF1 |  | isolated from citrus canker lesion in Florida |
